# The innovation of the clinical assistant role in a virtual emergency department: building digital health capability while supporting equitable access

**DOI:** 10.3389/frhs.2026.1872602

**Published:** 2026-06-30

**Authors:** Joanna Lawrence, Lizcha Kaivaha, Josua Lotter

**Affiliations:** Victorian Virtual Emergency Department, Northern Health, Epping, Australia

**Keywords:** digital navigation, socio-technical, sustainability, virtual care, workforce

## Abstract

The rapid expansion of virtual care has exposed gaps in workforce design, particularly at the intersection of digital literacy, access, and clinical efficiency. The Clinical Assistant (CA) role is an innovative response to these challenges within the Victorian Virtual Emergency Department (VVED), a statewide telehealth service providing urgent medical care via video consultation. This perspective shares the development, implementation and impact of the CA role, drawing on service data and existing literature. CAs provide real-time technical and navigational support to patients, mitigate digital access barriers, and redistribute administrative tasks away from clinicians. Early service data and staff perceptions suggest improvements in workflow efficiency, clinician experience and patient access, particularly among digitally vulnerable populations. In parallel, the role offers unique exposure for medical and allied health students, supporting the development of digital health capabilities and systems-based understanding. The CA model highlights the importance of integrating socio-technical support roles into virtual care systems and provides insights for future workforce redesign in digitally enabled healthcare.

## Introduction: virtual care expansion and workforce gaps

Virtual emergency care has emerged as a scalable response to increasing demand for urgent healthcare, workforce constraints and geographic inequities (1–3). While telehealth has demonstrated capacity to improve access and reduce health system costs, its implementation has also revealed structural limitations, particularly in workforce design and digital inclusion.

The Victorian Virtual Emergency Department (VVED) is a large-scale example of a digitally enabled urgent care model, providing 24-hour video-based emergency consultations across the state of Victoria, Australia (4, 5). Currently, the service manages up to 1,000 patient presentations per day with approximately 90% of patients supported to remain at home through remote assessment, investigations and prescribing (internal VVED data). Despite this success, the effectiveness of such models is highly dependent on patients' ability to access and navigate digital platforms. Call drop-outs, incomplete registrations, and failure to connect due to limited digital literacy, language barriers and technological challenges present significant risks to access, safety, and service efficiency (6–8).

With expansion of referral pathways, including Nurse-on-Call services and ambulance triage, it became evident that dedicated personnel were required to support patients with accessing the service. The innovative CA role was developed to provide real-time technical and navigational support to patients experiencing connection issues. Once created, other administrative tasks, previously performed by medical staff, were delegated further improving the cost-efficiency and sustainability of the service. Recruitment for the role was highly competitive due to the experiential learning opportunities for medical students which provide unique exposure to the internal operations of a large medical service and close working relationships with clinical staff.

This perspective provides an overview of the role and shares outcomes from routine service evaluation and quality improvement activities.

## Data sources and analysis

We include results from a clinician survey (*n* = 92/150, response rate 61.3%) and a Clinical Assistant survey (*n* = 33/40, response rate 82.5%) performed 12 months after implementation of the role (October 2024). Surveys were internally developed for service evaluation purposes and administered electronically via Redcap (Tables 1, 2). Invitation to participate was distributed through WhatsApp chats, Teams Channels, meetings and email. Participation was voluntary and responses anonymous with participation taken as implied consent. Questions were asked using a 5-point Likert scale with a space for free-text comments. Clinicians were asked an additional question which was ‘On average, how many minutes an hour do you feel the assistance of the Clinical Assistant has saved you?’ Quantitative responses were analysed using descriptive statistics and qualitative data reviewed for illustrative comments. Ethics approval for quality improvement was obtained from St Vincents Hospital HREC LRR 089/22.

**Table 1:** Clinician survey – questions and results.

Question	Responses
How have you found the quality of the work performed by the Clinical Assistants	Very good: 78 Good: 12 Acceptable: 2 Poor:0 Very Poor: 0
The introduction of Clinical Assistants within VVED has enabled me to focus more on patient care	Strongly agree: 61 Agree: 24 Neutral: 6 Disagree: 1 Strongly disagree: 0
I am supportive of the integration of Clinical Assistants in the VVED	Strongly agree: 75 Agree: 18 Neutral: 0 Disagree: 0 Strongly disagree: 0
The use of Clinical Assistants has improved my experience of working in the VVED	Strongly agree: 68 Agree: 19 Neutral: 3 Disagree: 2 Strongly disagree: 0
The use of Clinical Assistants has contributed to patient safety and wellbeing	Strongly agree: 48 Agree: 30 Neutral: 11 Disagree: 3 Strongly disagree: 0
I would recommend the use of Clinical Assistants across other healthcare settings	Yes: 84 Unsure: 8 No: 0

**Table 2:** Clinical Assistant survey - questions and results.

Question	Responses
I enjoy my role as a Clinical Assistant	Strongly agree: 14 Agree: 18 Neutral: 0 Disagree: 3 Strongly disagree: 0
I feel safe and supported	Strongly agree; 21 Agree: 10 Neutral: 1 Disagree: 1 Strongly disagree: 0
What skills have you developed during your role as a Clinical Assistant:	
Communication and interpersonal skills	30/33
Teamwork and collaboration skills	28/33
Time management and organisational skills	28/33
Use of technology	29/33
Awareness of roles and responsibilities of healthcare workers	26/33

## Identifying the problem: digital and operational barriers to access

Patients register for VVED through an online platform and are subsequently sent a link to a virtual waiting room on their mobile device. In the early phases of the service, a substantial proportion of patients experienced difficulty completing registration or joining the virtual platform. These challenges were particularly pronounced among older adults, residents of residential aged care facilities (RACFs), and patients with low digital literacy, consistent with other reports in the literature and raising concerns about widening health inequities (6–11).

Clinicians ready to undertake clinical assessment were frequently diverted to troubleshooting technical issues via telephone. This resulted in workflow fragmentation, increased waiting times, and inefficient use of highly trained clinical staff. Similar challenges have been reported in other telehealth settings, where technical barriers increase consultation time and reduce clinician satisfaction (12). With the introduction of external referral pathways, an additional challenge emerged: partially completed registrations that relied on patients to complete final steps independently. Patients who did not connect within required timeframes became known as “breach” calls, representing both an access failure and a potential safety risk.

These issues highlight a critical gap in the virtual care model, the assumption that patients can independently navigate digital systems.

## Development and evolution of the clinical assistant role

To address these challenges, VVED developed a novel role: the Clinical Assistant (CA). Initially piloted during a period of rising demand in the lead-up to Christmas, the role leveraged medical students on summer break to provide targeted technical and navigational support to patients. Early operational observations suggested reductions in clinician time spent on non-clinical tasks and improvements in patient flow.

As the role became embedded, its scope expanded organically in response to service needs (Table 3). CAs assumed responsibility for managing breach calls, supporting a significant proportion complete and providing appropriate alternative follow-up and escalation for those unable to connect. Additional responsibilities included chasing pathology and radiology results, facilitating discharge documentation, and communicating outcomes to hospitals, general practitioners, and community clinicians. These functions further reduced administrative burden on clinical staff.

**Table 3:** Functions of the clinical assistant role within the VVED.

Clinical assistant function	Problem addressed	Stakeholders	Expected outcomes
Registration support and patient onboarding	Incomplete registrations, difficulty navigating digital platforms; failure to access virtual care	Patients, carers, clinicians, referring services	Improved access to care, reduced digital exclusion, improved completion of virtual care pathways
Technical troubleshooting and virtual platform support	Connectivity issues, call drop-outs, low digital literacy, delayed consultations	Patients, carers, doctors, nurses	Reduced barriers to virtual care, improved consultation flow, reduced clinician time spent on technical issues
Management of breach and incomplete presentations	Patients failing to connect within required timeframes, potential delays in assessment and care	Patients, clinicians, referring services	Improved patient flow, reduced access failures, support for timely clinical assessment and escalation
Interpreter identification and coordination	Language barriers limiting equitable access to virtual care	Patients from CALD communities, interpreters, clinicians	Improved communication, enhanced equity of access, more efficient consultation processes
Collection of baseline information for GEDI and RACF patients	Time-consuming pre-assessment information gathering, fragmented information sources	Older adults, RACF residents, RACF staff, clinicians	Improved consultation efficiency, reduced administrative burden, support for earlier identification of clinical concerns
Investigation follow-up and results management	Delays locating pathology and radiology results, clinician administrative burden	Clinicians, external providers, patients	Improved workflow efficiency, reduced delays in decision-making, improved continuity of care
Patient, carer and service liaison	Communication gaps between patients, carers, clinicians and external services	Patients, carers, clinicians, RACF staff, referring services	Improved care coordination, improved patient experience, smoother transitions through care pathways
Digital navigation and health system guidance	Limited patient familiarity with telehealth systems and virtual care processes	Patients, carers, vulnerable populations	Reduced digital exclusion, increased patient confidence and engagement with virtual care
Student workforce participation and experiential learning	Limited opportunities for exposure to digital health systems and virtual models of care	Medical, nursing, paramedic and allied health trainees; health services	Development of digital health capability, improved systems-based understanding, workforce pipeline development

CA, clinical assistant; VVED, Victorian Virtual Emergency Department; GEDI, geriatric emergency department intervention; RACF, residential aged care facility; CALD, culturally and linguistically diverse.

Within the Geriatric Emergency Department Intervention (GEDI) workflow, the CA role was further adapted to support the needs of older patients and RACF residents. CAs collected baseline clinical information from RACF nursing staff prior to clinician assessment, including past medical history, medication lists, recent vital signs, and key contact details. By front-loading this information, clinicians were able to focus immediately on the presenting problem, improving consultation efficiency and supporting early identification of red flags in a population known to present atypically.

Over time, the CA workforce diversified to include medical, nursing, paramedic, and allied health trainees. Staff were selected based on strong communication skills, technical competence, and health literacy, reflecting the multidisciplinary and socio-technical nature of virtual emergency care.

## Related roles

Similar roles have emerged such as the digital navigator or telehealth coordinator to support access to digital care (13, 14). However, unlike many digital navigator roles described in outpatient or primary care settings, and focussed on supporting access to portals, the CA role is embedded within a high-acuity virtual emergency service requiring rapid trouble shooting, escalation awareness and close operational integration with clinicians. In particular, the CA role is distinctive in three aspects:

1. Real-time, pre-consult and intra-consult technical support integrated into the emergency care workload
2. Direct workload redistribution from clinicians with measurable time savings
3. Explicit workforce development mandate, embedding supervised exposure for medical students to virtual emergency pathways and digital health competencies.

## Supporting digital literacy and equitable access to care

Within VVED, CAs function as digital navigators. While the technological requirements for accessing the service are relatively simple, disparities in digital literacy create substantial barriers to care (8). By providing real-time technical support, assisting with registration, and proactively managing incomplete or breached presentations, CAs help ensure that access to urgent care is not determined by a patient's age, location, or digital confidence.

By offering step-by-step guidance and reassurance, CAs help patients unfamiliar with telehealth platforms develop competence and autonomy in navigating digital systems. This function is particularly important for individuals with limited prior exposure to digital technologies, and those from culturally and linguistically diverse (CALD) and marginalized communities.

## Impact on clinical efficiency and service sustainability

Task redistribution is a well-established strategy for improving healthcare efficiency (15). The CA role enables a redistribution of preparatory and administrative tasks away from doctors and nurses, allowing clinicians to operate at the peak of their scope of practice. By managing connection troubleshooting, incomplete registrations, breach calls, baseline data collection, and follow-up of investigations, CAs reduce workflow fragmentation in a high-demand environment.

Clinicians estimated that CAs saved on average 10-minutes per hour. This reclaimed time supports improved consultation flow, reduces cognitive load, and contributes to clinician wellbeing.

99% of VVED clinicians surveyed (*n* = 92/93) a year after commencement of the role (October 2024) agreed that workflows had improved. 92% reported being able to focus more on clinical tasks (*n* = 85/92) and 95% agreed or strongly agreed that it improved their experience of working in the VVED (*n* = 87/92). The quality of the work performed by CAs was considered of high or very high standard (*n* = 91/93, 98%).

Clinicians commented:

‘They are fantastic and such an asset to our team. When they are not on shift, it is very hard.’

‘They have made a huge impact on improving workflow and increasing clinical time with the patient’

‘Clin Assistants have made VVED a better place to work. I've found their enthusiasm to help, and positive attitude and desire to find solutions, gives our workplace something special. I hope that we are providing something valuable in return,’

‘As a Nurse Unit Manager in VVED I find the Clinical Assistants invaluable particularly in the area of troubleshooting patients and their technology issues and the Health Direct messages to patients in VVED. This task used to fall to us and as we have got busier it free us up to do other tasks.’

‘They are integral to the VVED GEDI program in both the support of the individual practitioners by reducing time spent on interventions with their scope of practice. They support the coordination of the service which has a flow through effect.’

## Workforce development and digital health capability building

From a workforce perspective, the CA role provides a unique immersion into virtual emergency medicine and the operation of a large healthcare system. Although primarily operational, the role offers continuous exposure to real-time clinical decision-making, including assessment, investigation, prescribing, and disposition across a high-volume and diverse caseload. This aligns with calls to embed digital health competencies in health professional education (16).

CAs observe clinician-to-clinician communication through internal messaging platforms, gaining insight into clinical reasoning and logistical coordination specific to virtual care. Within the GEDI workflow, this includes recognising subtle deterioration in older adults who may present with deceptively normal vital signs, supporting early development of clinical pattern recognition.

Exposure to referral pathways, community services, and discharge processes enhances systems-based understanding, a competency often underdeveloped in undergraduate medical, nursing, and paramedical education.

CAs reported enjoying the role (32/33, 97%), with 94% (*n* = 31/33) reporting feeling safe and supported. Skills reported to have improved through working as a CA included technical skills (*n* = 29/33, 88%), communication skills (30/33, 85%), teamwork (28/33, 82%), time management (28/33, 82%), awareness of roles and responsibilities of healthcare workers (26/33, 79%).

Medical student CAs have consistently reported that the role supports transition readiness for internship by combining clinical exposure with practical health system literacy. Many also describe the role as providing inspiration and clarity regarding future careers in health care.

## Limitations and implementation considerations

Several implementation considerations should be acknowledged when adopting and scaling the CA role. Clear governance structures and well-defined scope of practice are essential to ensure that clinical responsibilities are not unintentionally redistributed over time. As the role evolves in response to operational pressures, there is potential for “scope creep,” particularly in high-demand environments where CAs work closely alongside clinicians. Ongoing supervision, role delineation, and regular review processes are therefore necessary to maintain patient safety and ensure that CAs function within a clearly non-clinical or delegated support capacity.

Training and onboarding require significant organisational investment. CAs must be equipped not only with technical proficiency in telehealth platforms, but also with communication skills, escalation processes, privacy and confidentiality requirements, cultural awareness, and an understanding of virtual emergency workflows. Standardised training frameworks and competency assessment processes are important to ensure consistency and safety across the workforce, particularly as responsibilities expand.

The workforce model also presents operational challenges. Reliance on students and trainees creates a highly transient workforce with predictable turnover linked to academic calendars, examinations, and clinical placements. Availability is often concentrated to evenings and weekends, creating variability in workforce coverage and requiring ongoing recruitment, orientation, and supervision. Maintaining workforce continuity and organisational knowledge can therefore be resource intensive.

In addition, while early service evaluation suggests improvements in clinician workflow and staff experience, formal evaluation of patient-centred outcomes, health equity impacts, safety outcomes, and cost-effectiveness remains limited. The current findings are derived primarily from service-level quality improvement activities and staff surveys within a single health service, which may limit generalisability to other settings. Further multicentre and prospective evaluation would strengthen understanding of the role's broader impact and sustainability across different virtual care models.

Finally, the success of the role is closely linked to the maturity of the surrounding virtual care infrastructure. Services without established digital systems, escalation pathways, or operational leadership may face challenges integrating similar workforce models effectively. Careful adaptation to local context, patient population, and service demand is therefore likely to be required.

## Conclusion

The CA role addresses a critical socio-technical gap in virtual emergency care by supporting digital access and redistributing non-clinical tasks from clinicians. Early service evaluation indicates improved workflows and clinician experience. Formal assessment of patient outcomes, equity impacts and cost-effectiveness is a priority for future work.

As virtual models of care continue to expand, roles such as the CA will be essential in aligning service sustainability, workforce development, and equitable access to high-quality emergency care.

## Data Availability

The original contributions presented in the study are included in the article/Supplementary Material, further inquiries can be directed to the corresponding author.
